# Supplementary material for: RUNX1/RUNX1T1 mediates alternative splicing and reorganises the transcriptional landscape in leukemia
Source: Nat Commun. 2021 Jan 22;12:520. doi: 10.1038/s41467-020-20848-z (PMC7822815; doi:10.1038/s41467-020-20848-z)
Supplement: Supplementary file 1 — Supplementary Information [file 41467_2020_20848_MOESM1_ESM.pdf]

Supplementary Figures.

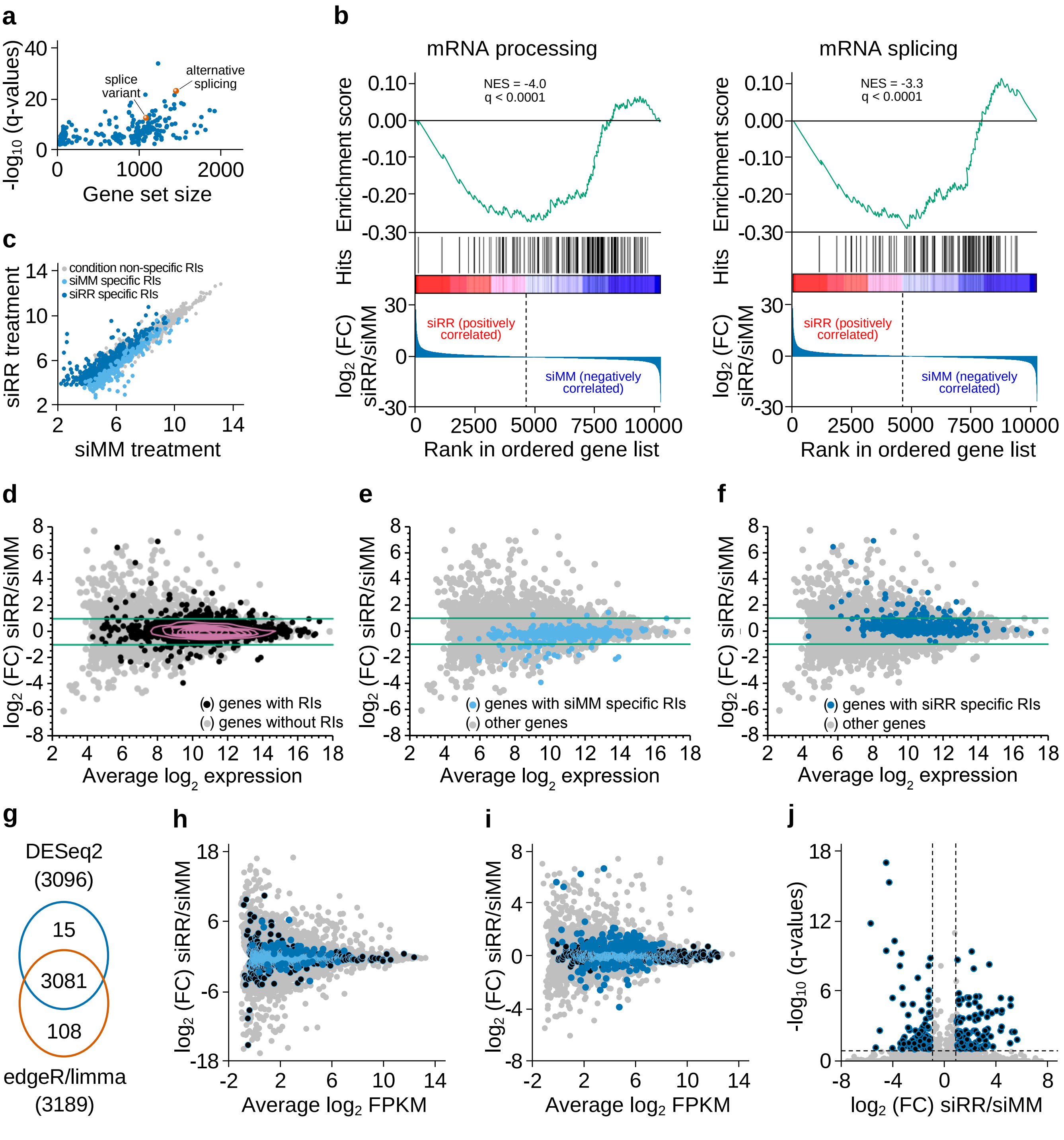

Supplementary Fig. 1 Knockdown of *RUNX1/RUNX1T1* affects exon usage in Kasumi-1 cells. **a** Scatterplot demonstrating DAVID results. **b** Gene set enrichment analysis plots for the Reactome mRNA processing and mRNA splicing pathways. NES, normalized enrichment score; q, false discovery rate. **c** Expression of condition-specific retained introns (RIs) in the siRR- and siMM-treated leukemia cells. The axes denote the normalized and log_2 transformed expression values averaged over the three independent biological repeats. Here and below, siMM-specific RIs are the RIs with statistically significant expression only in the siMM-treated leukemia samples. Similarly, siRR-specific RIs are the RIs with statistically significant expression in the siRR-treated but not in the siMM-treated leukemia samples.

d, e, f Series of diagnostic MA-plots of gene expression under the two siRNA treatment conditions. The genes that produce the RIs form relatively stable core of distribution. This core is bounded by a density contour in **d**. However, the genes that produce condition-specific RIs show a small but noticeable difference in expression. In these plots, bluish green lines indicate two-fold change in gene expression. Parts **c** to **f** are based on the analysis of the RIs detected using the DESeq2 algorithm. Very similar results were observed for the RIs detected by the edgeR/limma algorithm. **g** Diversity of the transcripts containing RIs detected using different approaches. For each approach, the total number of transcripts with RIs is indicated in parentheses. **h** Diagnostic MA-plot of three groups of transcripts: i) transcripts without RIs (●), ii) stably expressed transcripts containing RIs (◉), and iii) differentially expressed transcripts containing RIs (●). **i** Diagnostic MA-plot of three groups of genes: i) genes that produce transcripts without RIs (●), ii) stably expressed genes which produce transcripts with RIs (◉), and iii) differentially expressed genes which produce transcripts with RIs (●). **j** The JunctionSeq algorithm identified 221 diffUEs distributed over 142 individual genes.

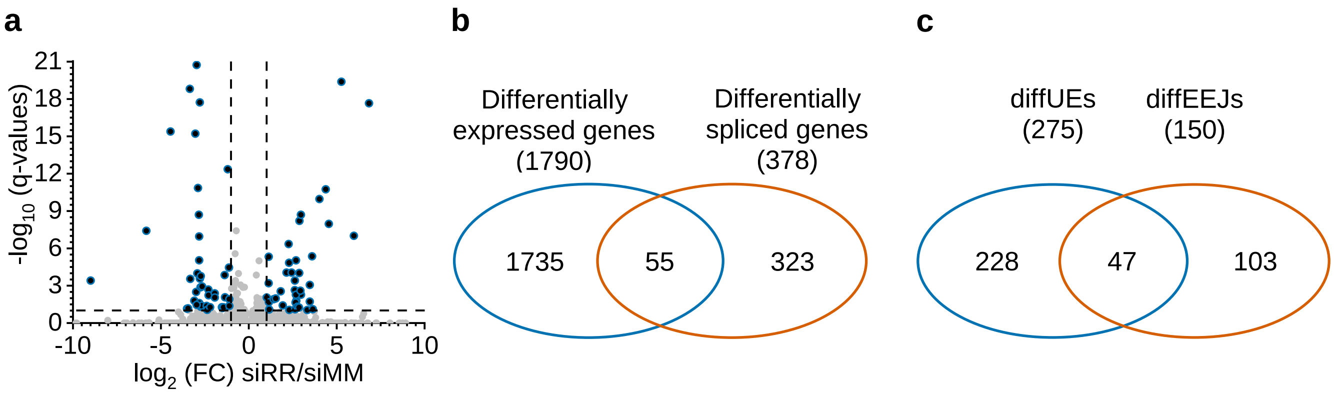

Supplementary Fig. 2 Knockdown of *RUNX1*/*RUNX1T1* causes changes in expression and splicing of the significant fraction of genes in Kasumi-1 cells. **a** Vulcano plot showing differential splicing of exons identified by JunctionSeq algorithm. Blue dots indicate differentially used exon-exon junctions (diffEEJs) with more than 2-fold change and $q < 0.1$. **b** Venn diagram demonstrating overlap between the lists of differentially expressed and differentially spliced genes upon RUNX1/RUNX1T1 knockdown. The list of differentially expressed genes was compiled based on the results of DESeq2, edgeR/limma and Cufflinks/Cuffdiff algorithms. Similarity, the list of differentially spliced genes includes genes for which differential splicing was found at the level of exons or exon-exon junctions (EEJs) using DEXSeq, limma/diffSplice and JunctionSeq algorithms. **c** Venn diagram showing overlap between the lists of genes with differentially used exons (diffUEs) and diffEEJs following RUNX1/RUNX1T1 knockdown. The list of genes with diffUEs is based on the results of DEXSeq, limma/diffSplice and JunctionSeq algorithms. The list of genes with diffEEJs was compiled based on the results of limma/diffSplice and JunctionSeq algorithms.

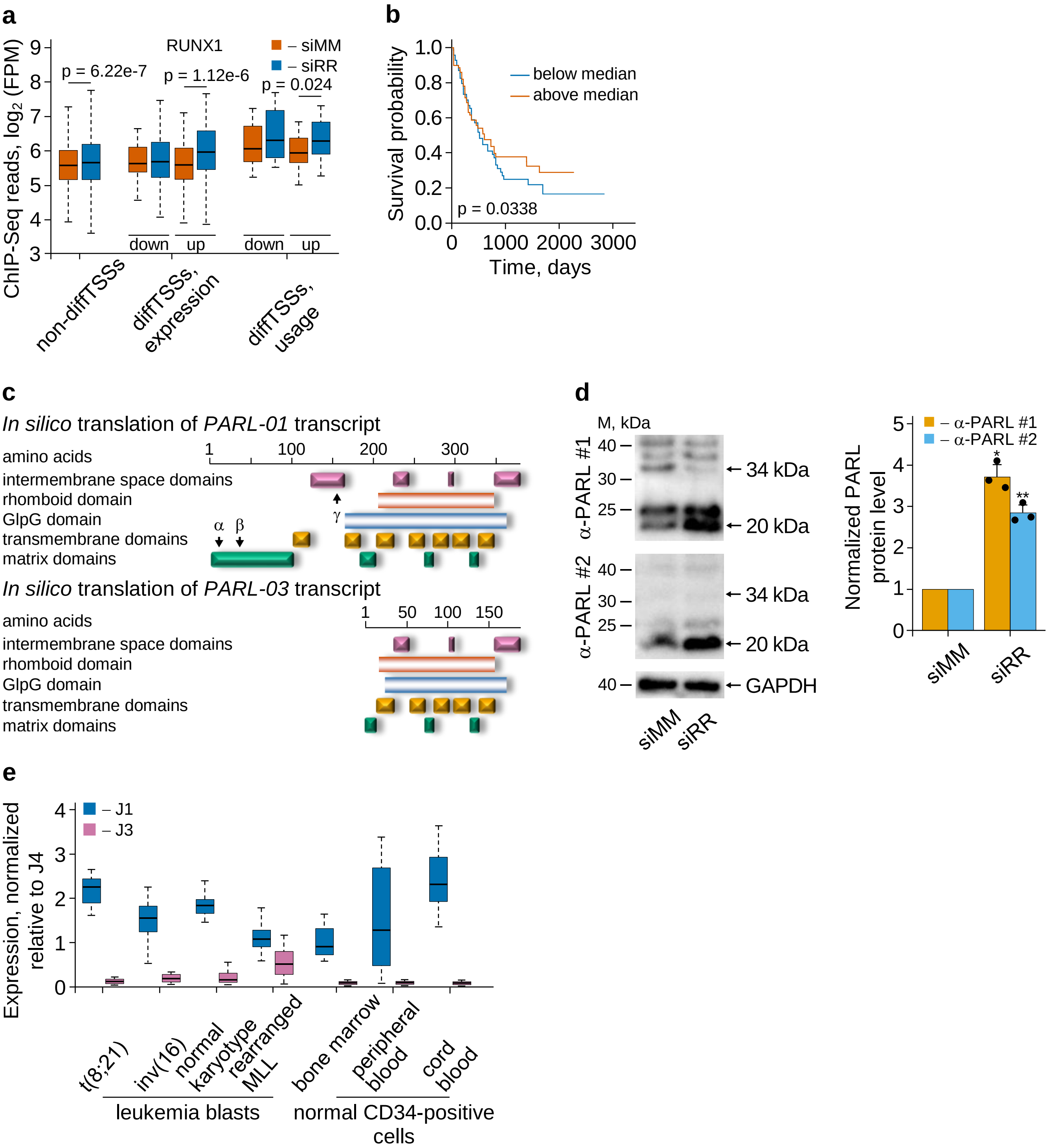

Supplementary Figure 3. RUNX1/RUNX1T1 controls alternative splicing of *PARL* transcripts in t(8;21)-positive leukemia cells. **a** Enrichment of RUNX1 ChIP-Seq reads in the genomic regions surrounding the non-differential TSSs, differentially expressed TSSs or differentially used TSSs. In this analysis, only TSSs genomic regions that overlap the RUNX1/RUNX1T1 binding peaks were used. In each boxplot, horizontal line represents the median of expression distribution, box shows the interquartile range, and whiskers are the minimum and maximum. (*) $p < 0.05$ and (**) $p < 0.0001$ with two-sided Mann-Whitney U test. **b** Survival of AML patients depending on expression of *PARL* gene. This survival plot is based on TCGA-LAML dataset (n = 173). The dependence of patient survival on gene expression was calculated according to the Cox proportional hazards regression model. **c** Domain

architecture of the *in silico* translated two PARL proteins. The cleavage sites of the full-length isoform of the PARL protein are marked by arrows. **d** Western blot (left panel) of PARL protein levels in Kasumi-1 cells following RUNX1/RUNX1T1 knockdown. Quantitation of PARL protein expression (right panel) upon RUNX1/RUNX1T1 knockdown. According to one-tailed one sample Student's t-test with $\mu_0 = 1$, the observed changes for 20 kDa PARL protein expression are statistically different at (*) p = 0.00386 and (**) p = 0.000333. On right panel, error bars represent mean ± SD of three independent experiments. **e** Expression of J1 and J3 junctions relative to junction J4 in various types of human primary leukemia blasts or normal CD34-positive cells. This panel is based on the results obtained from 20 samples for each type of primary cells. In each boxplot, horizontal line represents the median of expression distribution, box shows the interquartile range, and whiskers are the minimum and maximum.

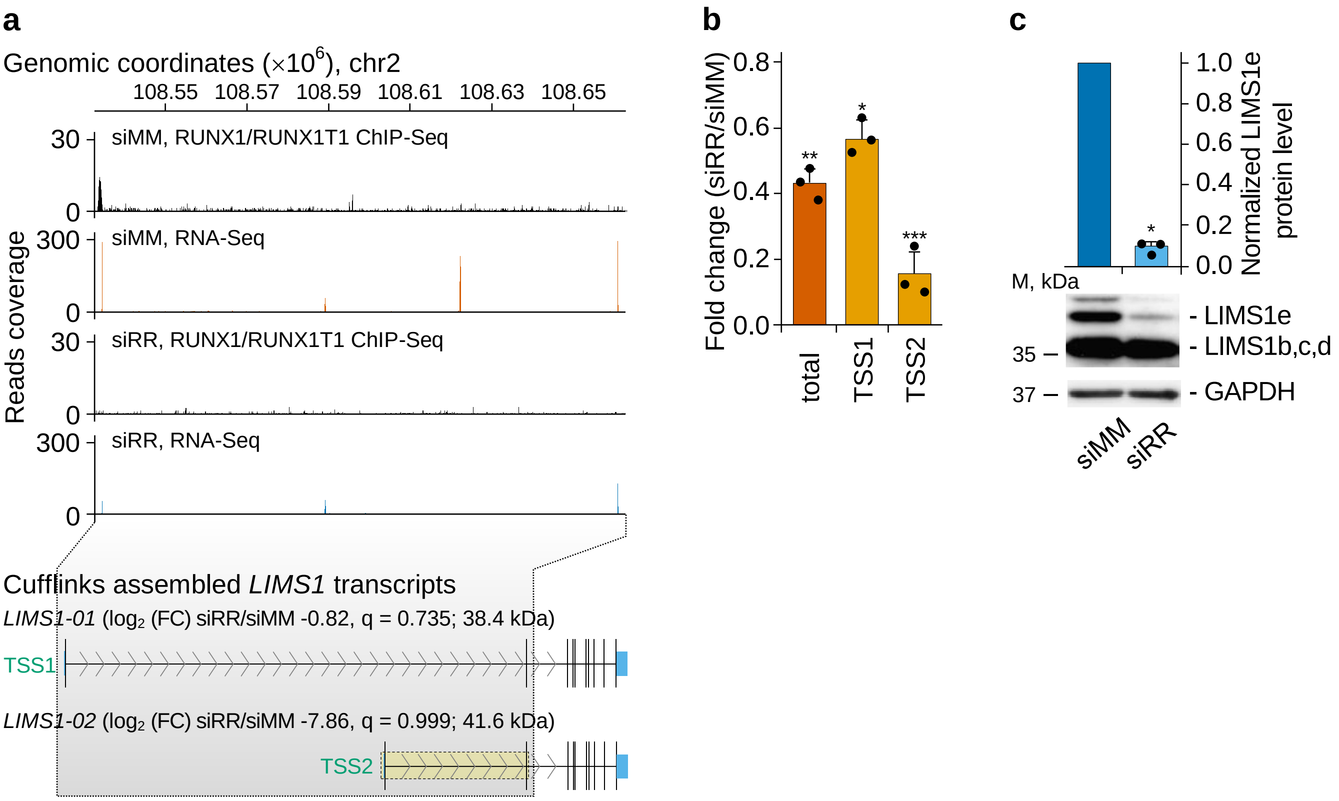

Supplementary Fig. 4 Knockdown of *RUNX1/RUNX1T1* leads to differential splicing of *LIMS1* transcripts in Kasumi-1 cells. **a** IGV screen shot demonstrating genomic coordinates, read coverage tracks and a set of representative Cufflinks assembled full-length transcripts. Read coverage tracks demonstrate the RUNX1/RUNX1T1 ChIP-Seq and total RNA-Seq results separately for siMM- and siRR-treated leukemia cells. Cufflinks transcripts are provided with Cuffdiff based $\log_2$ fold changes in expression and q-values as well as the size of *in silico* predicted proteins. The positions of the Cuffdiff determined transcription start sites are shown in green. In addition, limma/diffSplice identified exon-exon junction is boxed and highlighted in yellow. **b** qPCR-based quantitation of the change in the activity of various transcription start sites of *LIMS1* gene upon *RUNX1/RUNX1T1* knockdown. The overall expression level (“total”) of *LIMS1* gene is also indicated. According to one-tailed one sample Student’s t-test with $\mu_0 = 1$, the observed changes are statistically different at (*) p = 0.0053, (**) p = 0.0016 and (***) p = 0.0015. Error bars represent mean ± SD of three independent experiments. **c** Knockdown of *RUNX1/RUNX1T1* leads to change in the LIMS1 protein level in leukemia cells. Expression of the long isoform e of this protein changes the most dramatically (p = 0.00801 (*)). P-value was calculated using one-tailed one sample Student’s t-test with $\mu_0 = 1$. Error bars represent mean ± SD of three independent experiments.

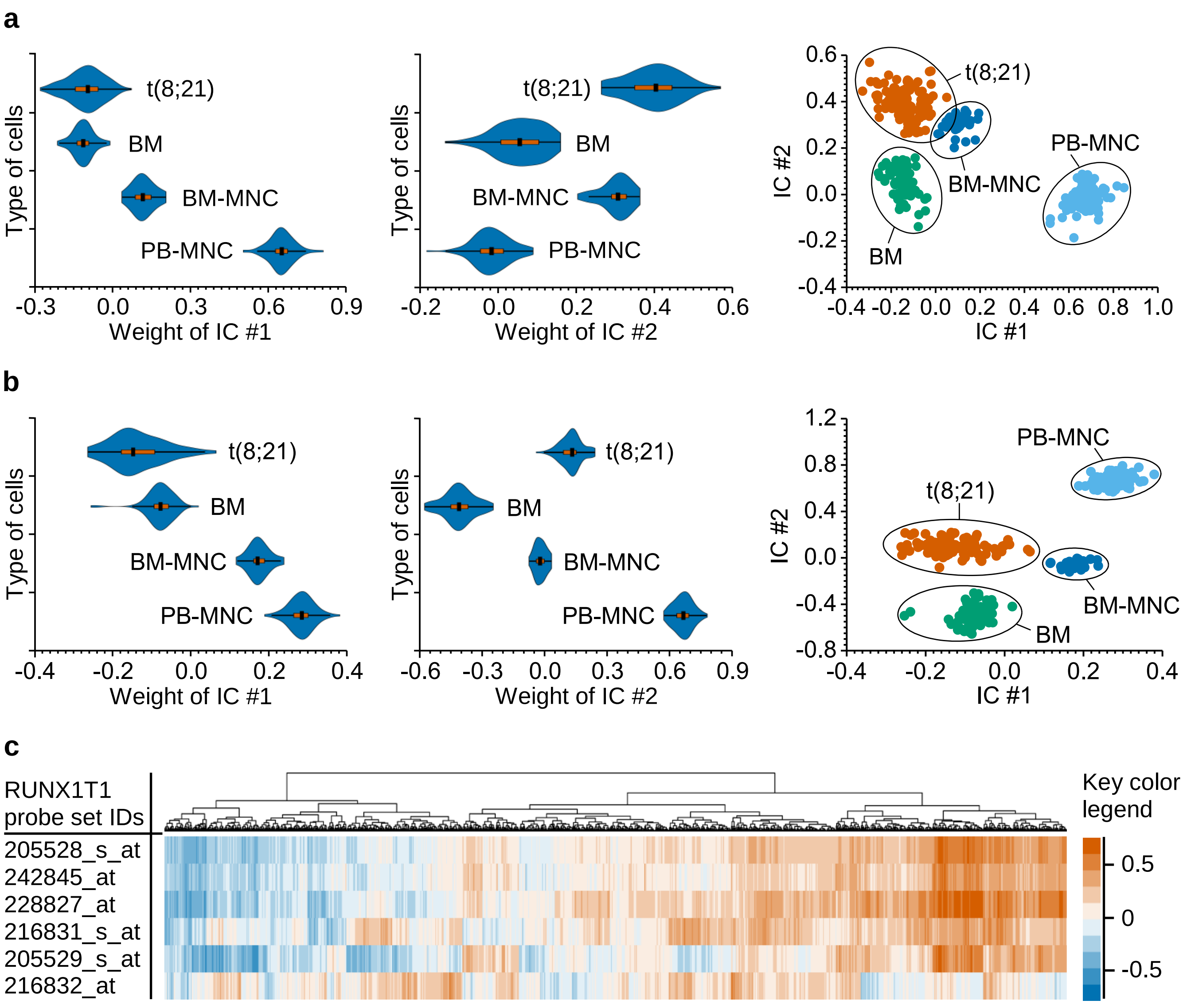

Supplementary Fig. 5 Independent component analysis identified a leukemia specific signature in the expression of genes encoding splicing factors and mRNA surveillance genes. This analysis was based on the public microarray data collected from 106 t(8;21)-positive AML samples (designated as t(8;21)), 85 samples of normal bone marrow (BM), 32 samples of normal bone marrow mononuclear cells (BM-MNC) and 100 samples of normal peripheral blood mononuclear cells (PB-MNC) using Affymetrix® HG-U133 Plus 2.0 gene chip. **a** Independent component analysis of the whole set of genes expressed in the samples of interest. This type of analysis revealed two subsets of genes that distinguish the t(8;21)-positive leukemia cells from the normal cells of hematopoietic origin (right scatter plot). These genes form independent component 1 (IC #1, 1681 genes, FDR < 0.05; left violin plot) and independent component 2 (IC #2, 1071 genes, FDR < 0.05; middle violin plot). One-way ANOVA test confirms the inequality of the means between the different types of cells ($p < 0.001$ for each of independent components). Moreover, according to the two-sided Fisher's exact test, each of the components is statistically significantly ($p < 0.001$) enriched with genes coding splicing factors and mRNA surveillance genes (1.62 and 1.66 fold enrichment for IC #1 and IC #2, respectively). In total, these independent components contain 150 genes encoding splicing factors and mRNA surveillance genes. **b** Independent component analysis of only a subset of splicing factor genes and mRNA surveillance genes expressed in the samples of interest. According to this type of analysis, t(8;21)-positive leukemia cells can be clearly

separated from normal cells of hematopoietic origin using only expression data of the genes encoding splicing factors and mRNA surveillance genes (right scatter plot). It is noteworthy that not all the genes encoding splicing factors and mRNA surveillance genes are required for this, but only two subsets of such genes that form two new independent components (violin plots). **c** Heatmap of the co-expression of *RUNX1*/*RUNX1T1* and the genes encoding splicing factors and mRNA surveillance genes in 106 t(8;21)-positive AML samples. The co-expression was inferred from microarray data by calculating the Pearson's correlation coefficient. In this co-expression analysis, the RUNX1T1 probe sets (left side of picture, rows) were used as specific indicators of *RUNX1*/*RUNX1T1* expression because *RUNX1T1* gene itself is not active in the t(8;21)-positive leukemia cells. The upper dendrogram (columns) represents the results of hierarchical clustering of genes encoding splicing factors and mRNA surveillance genes. Key color legend shows the range of values of the Pearson's correlation coefficient.

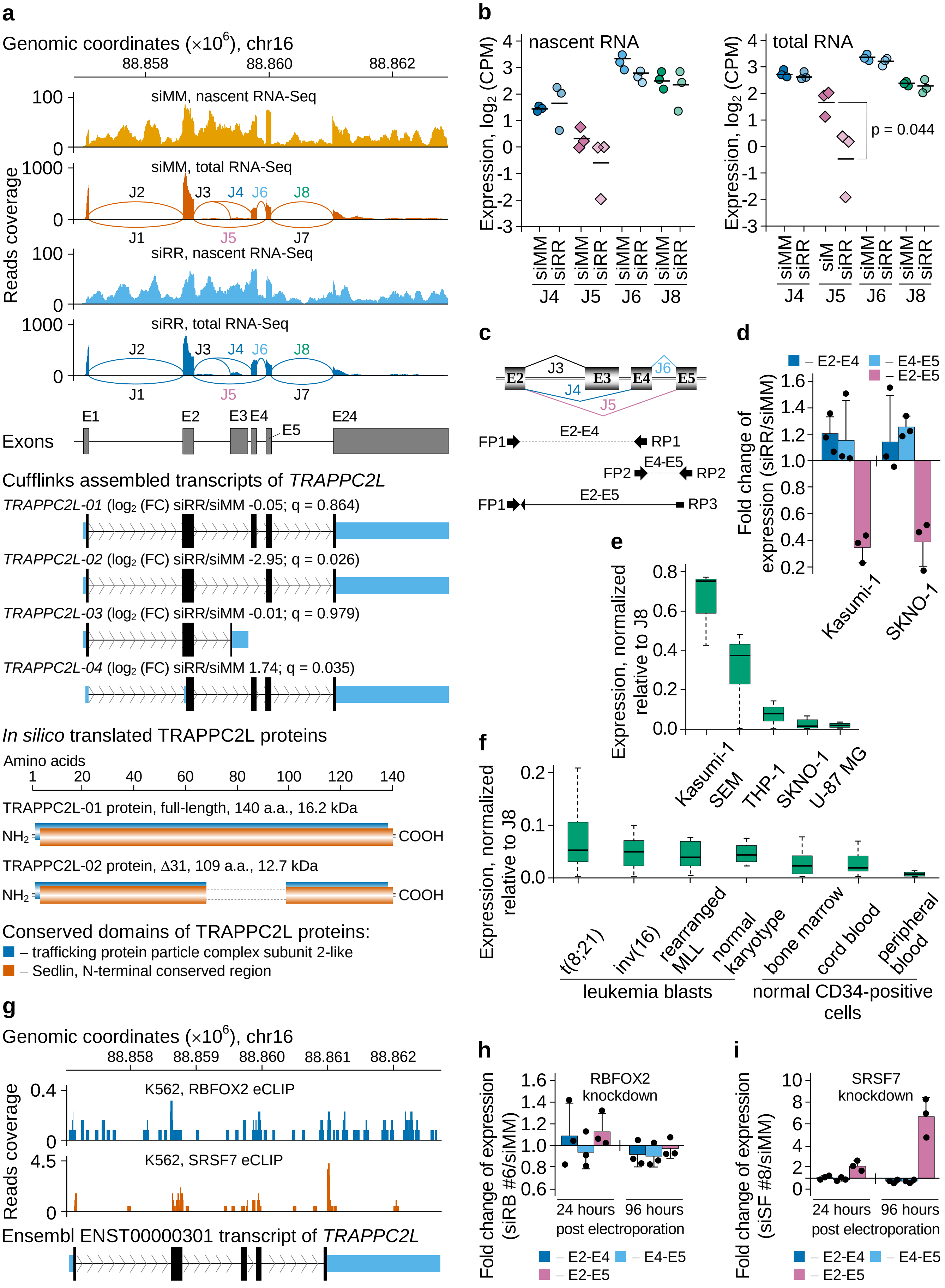

Supplementary Fig. 6 RUNX1/RUNX1T1 controls alternative splicing of *TRAPPC2L*

transcripts in t(8;21)-positive leukemia cells. **a** Splicing graphs, representative Cufflinks assembled transcripts and structure of two main *in silico* translated protein isoforms of *TRAPPC2L* gene. This panel is based on Kasumi-1 RNA-Seq data. Exons and exon-exon junctions are designated by the letters E and J, respectively, and numbered. Differentially used junction J5 is highlighted in purple. For each transcript, Cuffdiff based $\log_2$ fold changes in expression and respective q-values are indicated in parentheses. a.a. denotes amino acids. **b** Strip charts demonstrating normalized expression of exon-exon junctions J4 to J8 in Kasumi-1 cells. Genomic location of junctions is shown in **a**. Horizontal lines are arithmetic means of three independent experiments. P-value was calculated with two-sided Student's t test. **c** and **d** qPCR-based validation of the differential splicing of *TRAPPC2L* transcripts under two siRNA treatment conditions and in the transcriptome of two t(8;21)-positive cell lines. Binding positions of primers and structure of the expected amplicons are shown in **c**. Error bars in **d** represent mean ± SD of three independent qPCR experiments. **e** and **f** Expression of junction J5 relative to junction J8 in various types of human cell lines **e** and primary leukemia blasts or normal CD34-positive cells **f**. This panel is based on the results obtained from at least three independent measurements for cell lines and 20 samples for each type of primary cells. In each boxplot, horizontal line represents the median of expression distribution, box shows the interquartile range, and whiskers are the minimum and maximum. **g** RBFOX2 and SRSF7 proteins bind *TRAPPC2L* gene. This genome browser snapshot is based on ENCODE eCLIP data. **h** siRNA-mediated knockdown of the *RBFOX2* expression doesn't lead to significant differential splicing of the *TRAPPC2L* gene. **i** siRNA-mediated knockdown of the *SRSF7* expression leads to differential splicing of the *TRAPPC2L* gene. Error bars in **h** and **i** represent mean ± SD of three independent experiments.

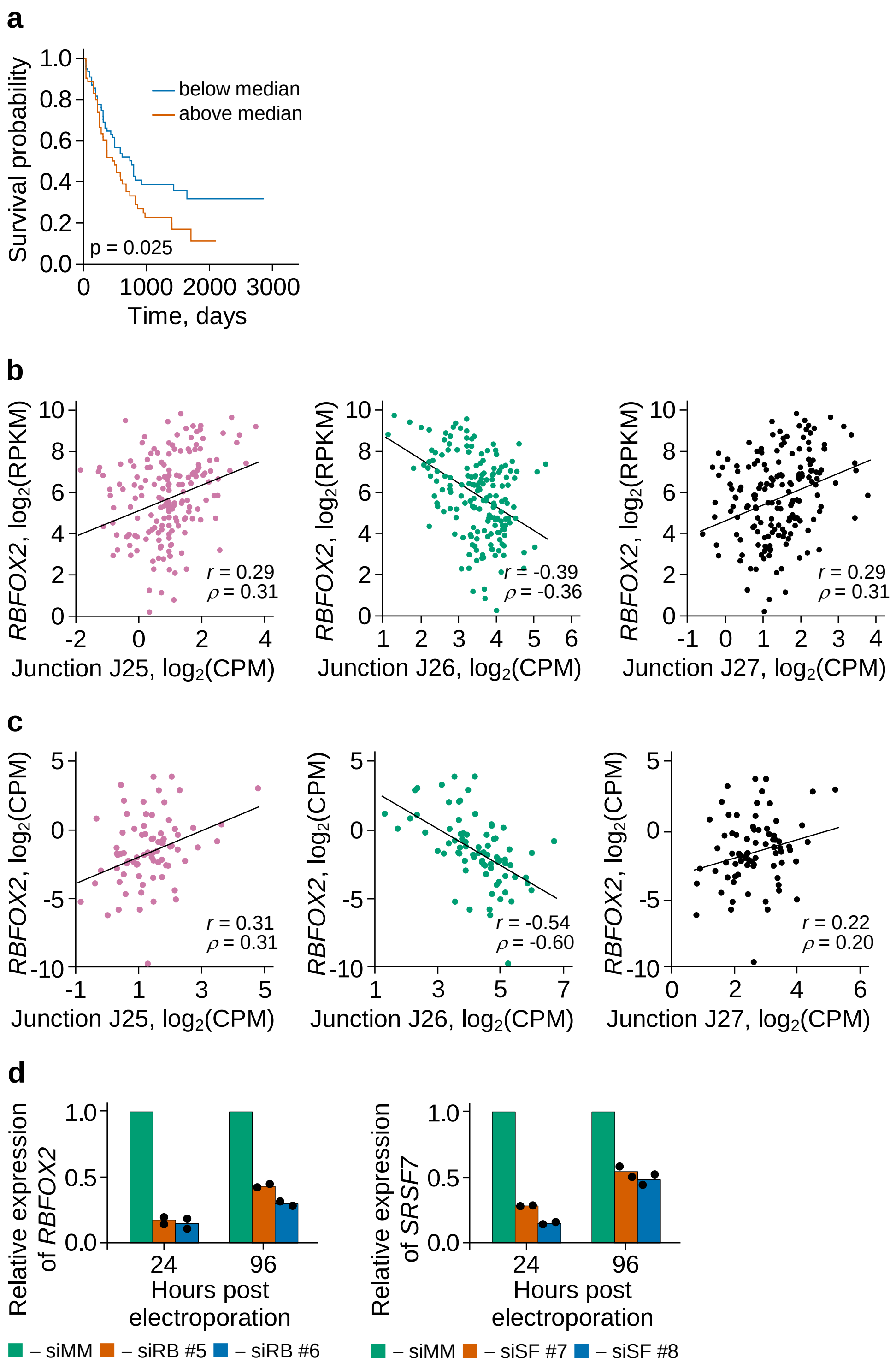

Supplementary Fig. 7 Expression of the *PTK2B* gene in leukemia cells. **a** Survival of AML patients depending on expression of the *PTK2B* gene. This survival plot is based on TCGA-LAML dataset (n = 151). The dependence of patient survival on gene expression was calculated according to the Cox proportional hazards regression model. **b** and **c** Scatterplots demonstrating correlation between expression of the *RBFOX2* gene and abundance of the *PTK2B* exon-exon junctions in leukemia blasts from TCGA-LAML dataset (n = 173) **b** and GEO/ENA-AML dataset (n = 77) **c**. **d** Efficiency of the *RBFOX2* and *SRSF7* knockdown by specific siRNAs from two independent experiments.

Supplementary Tables.

Supplementary Table 1: Abundance of EEJs identified in the nascent or total RNA from the siMM- or siRR-treated Kasumi-1 cells

Metric	nascent RNA				total RNA			
	non-diffEEJs		diffEEJs		non-diffEEJs		diffEEJs	
	siMM	siRR	siMM	siRR	siMM	siRR	siMM	siRR
Skewness	13.9	17.1	10.6	11.8	23.1	24.2	10.3	10.5
Kurtosis	404.1	751.1	122.7	144.4	727.9	802.7	113.3	118.2
1st quartile	1.3	1.3	0.0	0.0	2.0	2.0	0.5	0.4
Median	3.4	3.4	0.5	0.5	4.4	4.4	2.2	2.0
Mean	6.7	6.5	4.5	4.5	12.3	12.6	24.2	17.3
3rd quartile	7.5	7.5	1.9	2.1	9.9	9.7	5.9	6.6
Maximum	773.4	1072.5	288.3	367.9	2786.7	3429.3	1971.9	1248.0

Supplementary Table 2: siRNA sequences

Gene symbol	siRNA	Sense strand, 5’ → 3’	Antisense strand, 5’ → 3’
RBFOX2	siRB #5	UAAGAAGAUGGUCACACCAUA	UAUGGUGUGACCAUCUUCUUA
	siRB #6	CGGGUUCGUAACUUUCGAGAA	UUCUCGAAAGUUACGAACCCG
SRSF7	siSF #7	AAGGAUCGAGGUAUUUCCAAU	AUUGGAAAUACCUCGAUCCUU
	siSF #8	CAAGAUCUAUCUCUCUUCGUA	UACGAAGAGAGAUAGAUCUUG
RUNX1/RUNX1T1	siMM	CCUCGAAUUCGUUCUGAGAAG	UCUCAGAACGAAUUCGAGGUU
	siRR	CCUCGAAAUCGUACUGAGAAG	UCUCAGUACGAUUUCGAGGUU

Supplementary Methods

Detailed analytical approach

Quality assessment and pre-processing of the RNA-Seq raw data. A comprehensive RNA-Seq data quality assessment and pre-processing of the raw data was performed using the R/Bioconductor library ShortRead v.1.38.0 and the standard R infrastructure [1].

Alignment of the short RNA-Seq reads against the reference genome. GRCh38/hg38 reference assembly of the human genome was downloaded as twoBit file from the FTP server of the UCSC Genome Browser. It was then converted to a standard FASTA format with twoBitToFa utility [2]. A hash table for the reference genome was built with the function buildindex from the R/Bioconductor library Rsubread v.1.22.3 [3]. At this step, 16-mers subreads were extracted in every 3 bases from the reference genome and the threshold 24 was used to exclude highly repetitive subreads from the created hash table.

A global alignment of RNA-Seq reads against the reference genome was carried out with the function subjunc from the R/Bioconductor library Rsubread v.1.22.3 and the created hash table. This function implements a seed-and-vote mapping paradigm for a fast and accurate alignment [3]. At this step, we used the default settings of the function subjunc and collected only uniquely mapped reads with the maximum of 3 mismatched bases in the alignment. The resulting BAM files were sorted with the function sortBam and indexed with the function indexBam both from the R/Bioconductor library Rsamtools v.1.24.0 [4].

Development a list of non-overlapping genomic bins (fragments). First of all, annotations of the human genes were downloaded from the Ensembl database. These annotations were converted into an object of a class TranscriptDb and saved as a local SQLite database. All the subsequent manipulations with genomic intervals were performed using the genomic ranges infrastructure [5].

Next, the genomic coordinates of the annotated retained introns were calculated as follows. For each gene, the genomic coordinates of exons and introns were extracted from a TranscriptDb object and intersected using the function findOverlaps from the R/Bioconductor library GenomicRanges v.1.32.3 [6]. The intronic intervals that completely fell into the coordinates of exons of the same gene were selected. These intervals were subsequently intersected with the remaining exons of the gene and, if necessary, disjoined and dropped out due to overlapping. Additionally, the intervals shorter than 100 nucleotides or duplicated intervals were removed from the final list. We called these intervals annotated retained introns, since they are already present in the Ensembl annotation database. Moreover, we further sub-divided these introns into four sub-groups: i) annotated retained introns that completely fall into the alternative first exon (AnnoRI_FIRST), ii) annotated retained introns that are flanked by the internal exons (AnnoRI_INTERNAL), iii) annotated retained introns that completely fall into the alternative last exon (AnnoRI_LAST), and iv) introns that can be inferred as retained because of the overlap with one-exon transcripts (AnnoRI_OneExonTranscript).

Third, the function intronicParts of the R/Bioconductor library GenomicFeatures v.1.32.0 was used to extract non-overlapping intronic bins from a TranscriptDb object [7]. Extracted intronic bins were disjoined and dropped out against exons of one- and multi-exons genes (including genes of rRNAs, tRNAs, miRNAs, miscRNAs, ribozymes, vaultRNAs, sRNAs, snRNAs, scaRNAs, scRNAs and snoRNAs). Additionally, any intronic bins shorter than 100 nucleotides were removed. We called these intronic bins canonical introns. The final list of such introns was extended with the annotated retained introns, sorted, indexed, assigned with genes information and converted into an object of a class GRanges.

Fourth, the function exonicParts of the R/Bioconductor library GenomicFeatures v.1.32.0 was used to extract non-overlapping exonic bins from a TranscriptDb object [7]. Extracted genomic bins were disjoined and dropped out against genomic coordinates of the annotated retained

introns, and genomic bins shorter than 10 nucleotides were removed. The final list of the genomic bins was sorted, indexed, annotated with genes information and converted into an object of a class GRanges.

Finally, all the above-mentioned GRanges objects were joined into GRangesList and used in the downstream analysis.

Development a list of exon clusters. For each gene, the genomic coordinates of exons were retrieved from Ensembl annotations. These coordinates were intersected and joined into overlapping groups called exon clusters. The exon clusters shorter than 10 nucleotides were removed, and the final list of genomic intervals was sorted, assigned with genes information and converted into an object of a class GRanges.

Read summarisation. We used the function featureCounts from the R/Bioconductor library Rsubread v.1.22.3 to assign the mapped RNA-Seq reads to the genomic features in case of exonic and/or intronic bins or to the meta-features (genes) in case of exon clusters [3,8]. Each read pair was counted in unstranded mode with the minimum 1 base overlapping an exonic bin or exon cluster and minimum 5 bases overlapping an intronic bin. Herewith only one end of the read pair was required to be successfully aligned before the read pair was assigned to a feature or meta-feature.

In silico identification of retained introns. First, the primary count matrix of intronic bins was loaded into the R workspace and an effective length for each intron was calculated using the wgEncodeCrgMapabilityAlign100mer mapability table from the UCSC Genome Browser [2,9]. During this step, positions of the non-unique 100-mer alignments and 5 nucleotides from each end of the intron were summed and then subtracted from the original intron's length to produce mapability-adjusted intron length.

Second, the primary count matrix of the intronic bins was filtered against the non-expressed genes, intron effective length less than 100 nucleotides and one-bin genes. Third, a variance stabilizing transformation based on the square root of the intron effective length adjusted by the RNA-Seq read length was used to weight individual introns [10,11]. The sum of intronic reads per gene in each RNA-Seq sample was then partitioned and allocated to each intron proportional to its weight. This led to an *in silico* null model sample, one corresponding to each of the original RNA-Seq samples.

Fourth, differential analysis was carried out to determine introns enriched in the observed reads compared to the *in silico* expected reads (if all the introns within a gene are present at equal levels). We used standard DESeq2 and edgeR/limma pipelines at this step [12-14]. Herewith, we discretized the null distributions for the first approach, since DESeq2 uses negative binomial generalized linear modelling, and we loaded the null distributions as it they are for the edgeR/limma pipeline.

Finally, the results of the differential analysis were parsed and filtered. We selected only introns that passed a false discovery rate (FDR) adjusted p-value threshold of 0.01, fold change threshold of 2 and a required minimum of 20 reads per 100 nucleotides of the effective length of an intron averaged over all the original RNA-Seq samples. These introns were called *in silico* detected retained introns, or simply retained introns. The primary count matrix of intronic bins was then reduced to a list of retained introns and it was added to the primary count matrix of the exonic bins.

Identification of differentially used exons (diffUEs) with DEXSeq. First, the primary count matrix of the exonic bins was loaded into the R workspace and filtered against non-expressed genes, one-bin genes and too low sequencing depth (fragments per million, or FPM, ≥ 1). The filtered count matrix was subsequently used to create a flattened GTF file and it was wrapped (together with a flattened GTF file, sample annotations and experimental design) into an object of a class DEXSeqDataSet [15].

Second, the size of each RNA-Seq library was normalized using the "median ratio method" and dispersion estimates were obtained using the function estimateDispersions from the

R/Bioconductor library DESeq2 v.1.20.0 [12,16,17]. Third, the diffUEs were determined using the functions testForDEU and estimateExonFoldChanges from the R/Bioconductor library DESeq2 v.1.20.0 in the default mode. At last, the final results were summarized using the function DEXSeqResults from the R/Bioconductor library DESeq2 v.1.20.0.

Identification of diffUEs with function diffSplice. First, the primary count matrix of exonic bins was subjected to filtering against the non-expressed genes, one-bin genes and too low sequencing depth (counts per million, or CPM, $\geq$ 1) and it was wrapped (together with the sample information) into a DGEList object [18]. Second, to calculate effective sizes of RNA-Seq libraries, the scaling factors were estimated using the "trimmed mean of M-values" method [19]. Third, by applying the calculated scaling factors, the count data were converted into CPM and logarithmically transformed, the mean-variance relationship was estimated, and the appropriate observational-level weights were calculated using the voom algorithm [20].

Fourth, the multiple simple linear models were fitted to the normalized count matrix by least squares method using the function lmFit from the R/Bioconductor library limma v.3.36.1 [13,21]. Fifth, contrast coefficients (logarithms for base two of fold changes, or $\log_2$ FC, between the treatment conditions) were calculated and loaded into the function diffSplice [13,21]. This function calculates the difference between the $\log_2$ FC for a given exon versus the average $\log_2$ FC for all the other exons for the gene of interest. In other words, this function tests for differential usage of exons for each gene and for each treatment condition. Finally, from moderated t-statistics, p-values were adjusted for multiple testing [22].

Identification of diffUEs using the functionality of the JunctionSeq library. First, the overall quality of the BAM files was assessed with Picard v.2.9.0 (http://broadinstitute.github.io/picard/) and low-quality reads were removed using in-laboratory developed R code. Second, the flattened GFF file was created using toolset QoRTs [23]. This file was based on the Ensemble annotations of the human genome and included all the exons, annotated and novel exon-exon junctions (EEJs). Third, reads counts were generated by QoRTs. At this step, we counted all the reads mapped to exons, annotated or novel EEJs with minimum mapping quality of 30, and only events with minimum mean normalized read coverage of 10 were selected for downstream analysis.

Fourth, the diffUEs were identified by the sequential application of two functions runJunctionSeqAnalyses and writeCompleteResults in the default mode to reads counts. These functions are part of the R/Bioconductor library JunctionSeq v.1.10 and they use DEXSeq statistical infrastructure to detect diffUEs [15,24]. Finally, output results were parsed and adjusted to the formats of DEXSeq and diffSplice outputs by in-laboratory developed R code.

Functional classification of exons. Genomic coordinates of the reference exons were extracted from the Ensembl models of the human genes. These exons were grouped into five functional classes: 5'UTR exons, CDS exons, 3'UTR exons, exons of non-coding RNAs (NC) and multi-type exons (MTE) (exons that can be non-coding, 5'UTR, 5'UTR/CDS, CDS, CDS/3'UTR and/or 3'UTR exon depending on transcript). Next, each exonic bin was intersected with reference exons and was assigned to a functional class.

Identification of EEJs. All possible variants of EEJs were identified according to *Liao et al.* [3]. The resulting BED files were parsed and converted into the primary count matrix of EEJs with an in-laboratory developed R code. This matrix included a full list of identified EEJs with the respective genomic coordinates and a number of reads supporting each exon-exon junction in every sample.

Identification of the differentially used EEJs (diffEEJs) with function diffSplice. The primary count matrix of EEJs was subjected to filtering against too low sequencing depth (CPM, $\geq$ 1) and it was wrapped (together with the sample information) into a DGEList object [18]. All the subsequent steps of the analysis were carried out as described in section "Identification of diffUEs with function diffSplice", but at the level of EEJs.

Identification of diffEEJs using functionality of JunctionSeq library. Inferring of diffEEJs using the JunctionSeq library was performed as described in section “Identification of diffUEs using the functionality of the JunctionSeq library”, but at the level of EEJs.

Classification of EEJs according to the modes of alternative splicing. Our classifier of EEJs is based on the idea of hypothetical “non-alternative” precursor of RNA, or hnapRNA. hnapRNA is an RNA molecule that would have turned out if the gene had only one transcription start site (TSS), if there were no alternative splice sites, if there was no alternative splicing and if there was only one transcription termination site. In other words, hnapRNA is a generalization of all the RNA isoforms produced by the gene.

For each gene, the structure of the hnapRNA was calculated using Ensembl models of human genes. We clustered exons of the gene of interest into overlapping groups with the exception of retained introns, alternative 5’ and/or 3’ terminal exons. The outer boundaries of the resulting exon clusters were recorded as genomic coordinates of exons of the hnapRNA. The list of these coordinates was extended with coordinates of retained introns, alternative 5’ and/or 3’ terminal exons and it was converted into an object of a class GRanges.

Next, the genomic coordinates of EEJs were intersected with the coordinates of the features of the hnapRNA, and the mode of each EEJ was determined. According to our approach, all the EEJs were classified into eight modes of alternative splicing: i) canonical event, if the coordinates of the empirical event exactly match the model event; ii) alternative 5' splice site, if only the 3’ splice site of the empirical event exactly matches the respective model site; iii) alternative 3' splice site, if only the 5’ splice site of the empirical event exactly matches respective model site; iv) alternative both splice sites (intron isoform), if both splice sites of the empirical event do not match splice sites of respective model event; v) skipped cassette exon(-s), if the empirical event includes skipping one or more exons of the model; vi) alternative first exon, if the 5’ splice site of the empirical event exactly matches the 3’ end of alternative first exon in model; vii) alternative last exon, if the 3’ splice site of the empirical event exactly matches the 5’ end of alternative last exon in model; viii) complex splicing event, if the empirical event includes two or more of the above-mentioned alternative splicing events.

Reference-based transcriptome assembly. First, for each sample of RNA, we used Cufflinks and the respective subjunc-generated BAM file to assemble the alignments into a parsimonious set of transcripts [25,26]. Herewith, Cufflinks was supplied with i) Ensembl annotation of the human genome to guide RABT assembly, ii) a GTF file containing annotated human rRNA and mitochondrial genes to mask these genomic features during estimation of transcripts abundance, iii) complete sequence of the human genome in multiFASTA format to bias correction during the estimation of transcripts abundance, and iv) a minimal isoform fraction threshold assigned to 0.05.

Second, individual Cufflinks assembled transcriptomes were merged into one consolidated set of transcripts with Cuffmerge [27]. This set of transcripts was filtered against i) unstranded transcripts, ii) too short transcripts (<300 nucleotides), iii) transcripts with too short exon(-s) (<25 nucleotides), iv) transcripts with too short intron(-s) (<50 nucleotides), and vi) transcripts with low abundance (fragments per kilobase of transcript per million mapped reads, or FPKM, below 1). Filtration was controlled by in-laboratory developed R code.

Third, the consolidated and filtered set of transcripts was submitted to Cuffdiff for the simultaneous calculation of the transcript abundance and differential expression [28]. Cuffdiff was provided with a GTF file containing annotated human rRNA and mitochondrial genes and a multiFASTA file with complete sequence of the human genome, and it was run in default mode except for the minimal isoform fraction threshold that was assigned to 0.05. Finally, for the fast retrieving of the data and easy subsequent manipulations, the main outcomes of Cuffdiff were parsed, converted into an object of a class TranscriptDb and saved as a local SQLite database [29].

Identification of differentially expressed genes with Cuffdiff. We used Cuffdiff differential expression tests data (see section "Reference-based transcriptome assembly") to identify differential expression at transcript or gene levels between short interfering RNA treatment conditions. Herewith, only transcripts or genes with at least 2-fold changes in expression and q-value below 0.1 were annotated as differentially expressed.

Identification of differentially expressed genes with DESeq2. First, the mapped RNA-Seq reads were assigned to the genomic meta-features (genes) as described in section "Read summarisation". Second, the resulting count matrix was subjected to filtering against too low sequencing depth and it was wrapped (together with the sample information) into a DESeqDataSet object [16]. Third, differentially expressed genes were identified using the functions DESeq and results from the R/Bioconductor library DESeq2 v.1.16.1 [16]. These functions were run in default mode and according to the standard DESeq2 pipeline. Finally, results were parsed and genes with at least 2-fold changes in expression and q-value below 0.1 were annotated as differentially expressed.

Identification of differentially expressed genes with edgeR/limma. First, the mapped RNA-Seq reads were assigned to the genomic meta-features as described in section "Read summarisation". Second, the resulting count matrix was subjected to filtering against too low sequencing depth and it was wrapped (together with the sample information) into a DGEList object [18]. Third, to calculate an effective size of each RNA-Seq library, the scaling factors were estimated using the "trimmed mean of M-values" method [14]. Fourth, by applying the calculated scaling factors, the count data were converted into CPM and logarithmically transformed, the mean-variance relationship was estimated, and the appropriate observational-level weights were calculated using the voom algorithm [20].

Fifth, the multiple simple linear models were fitted to the normalized count matrix by least squares method using the function lmFit from the R/Bioconductor library limma v.3.36.1. Sixth, $\log_2$ FC coefficients and empirical Bayes statistics were calculated using respective functions from R/Bioconductor libraries edgeR v.3.22.3 and limma v.3.34.9 [14,30]. Finally, results were parsed and genes with at least 2-fold changes in expression and the q-value below 0.1 were annotated as differentially expressed.

Identification of differentially expressed and differentially used TSSs. Differentially expressed TSSs were identified according to the Cuffdiff algorithm [28]. Differential usage of TSSs was analysed according to idea implemented in diffSplice function of the R/Bioconductor library limma v.3.34.9 for each gene containing alternative TSSs.

Linear approximation of datasets using a principal component analysis (PCA). A standard PCA on the given data matrix was performed using the basic R function prcomp. This function was provided with centred and scaled data. Alternatively, multigroup PCA and/or kernel PCA were carried out using the R library "multigroup" v.0.4.4 and "kernlab" v.0.9-26, respectively [31,32]. The results of the linear approximations were used to calculate the "explained" variability and visualisation of the multidimensional data in the space of the first two or three principal components.

Linear approximation of datasets using a t-distributed stochastic neighbor embedding (t-SNE). A t-SNE was used as an alternative to the PCA where a deeper linear approximation of the data matrix was necessary. We used an R wrapper Rtsne for the Van der Maaten's C++ implementation of the Barnes-Hut algorithm of t-SNE [33,34]. This wrapper was run in the default mode except for perplexity and theta values, which were sequentially adjusted.

Linear approximation of datasets using an independent component analysis (ICA). An ICA was run on the combined microarray or RNA-Seq datasets for unsupervised separation of cell types and extraction of cell specific genes or EEJs. To perform analysis, an R wrapper fastICA for the Aapo Hyvärinen's implementation of the FastICA algorithm [35] was used in the default mode. The set of the most significant genes or EEJs per independent component was detected at FDR < 0.05 using previously described pipeline [36].

Identification of direct targets of RUNX1/RUNX1T1 fusion protein. We reanalysed our previous ChIP-Seq data to identify RUNX1/RUNX1T1 binding peaks in the genome of Kasumi-1 cells [37]. We used GSM722718, GSM722706 and GSM722707 FASTQ files for input, siMM- and siRR-treated Kasumi-1 cells. Reads were aligned against GRCh38/hg38 reference assembly of the human genome as described in section "Alignment of the short RNA-Seq reads against the reference genome". At this step, the function align was used instead of the function subjunc from the R/Bioconductor library Rsubread v.1.22.3 [3].

ChIP-Seq peaks were called in a frame of a fully Bayesian hidden Markov model with 10,000 Monte Carlo runs for each Markov chain [38]. Next, the peaks with more than 99.9% posterior probabilities were selected and depth/coverage filtered. The genomic coordinates of the final peaks were intersected with coordinates of the human genes and direct targets were identified. A gene was annotated as a direct target of the fusion protein if it has RUNX1/RUNX1T1 peaks within its transcription unit or in the immediate vicinity, $\leq$ 3,000 bp upstream of the first transcription start site.

Development a list of features associated with EEJs. Every exon-exon junction was annotated with sequence, sequence-related, functional, and structural features that were extracted from four types of genomic/RNA elements: 100-bp fragment of the upstream exon (USE), 300-bp fragment from the 5' end of the intron (USIF), 300-bp fragment from the 3' end of the intron (DSIF), and 100-bp fragment of the downstream exon (DSE). Additionally, each exon-exon junction was described with a set of nearest epigenetic marks. In total, the complete list of features included 1,680 items.

Splice sites scoring. Genomic coordinates of the 5' and 3' splice sites were retrieved from the matrix of the experimentally identified *EEJs* and sequences of these sites were extracted from the GRCh38/hg38 reference assembly of the human genome with the R/Bioconductor library BSgenome.Hsapiens.UCSC.hg38 [39]. Position weight matrices for the 5' splice sites (9-nucleotide sequence: 3 nucleotides in the exon and 6 nucleotides in the intron) and 3' splice sites (23-nucleotide sequence: 3 nucleotides in the exon and 20 nucleotides in the intron) were downloaded from the MIT MaxEnt Splice Site Scoring Server [40]. The strength of splice sites was determined by Perl implementation of the MaxEntScan algorithms in accordance with the three scoring models: maximum entropy model, first-order Markov model, and weight matrix model. The overall score of the splice sites for a given exon-exon junction was calculated as the sum of individual 5' splice site and 3' splice site scores averaged over the three models.

Experimentally verified exonic splicing motifs. We collected sequences of experimentally verified binding sites that were recognized by splicing-related proteins shown in Supplementary Table 5. For each protein and the respective set of sequences, we performed a motif search by the discriminative motif discovery algorithm motifRG [41] against the background set of randomly extracted human intronic and exonic sequences. The primary motif was refined by the function refinePWMMotif from the R/Bioconductor library motifRG v.1.18.0 with the default settings and was converted into the $\log_2$ position weight matrix with the correction against the background nucleotides frequency. Occurrence of a motif in the USE and DSE was determined by the function countPWM from the R/Bioconductor library Biostrings v.2.42.0 [42] and was normalized relative to the length of the analysed sequences. We used the 99th quantile of the motif weight distribution as a threshold in the identification of the true motif occurrence.

Additionally, we collected oligomeric sequences that were bound by splicing proteins. We were not able to calculate position weight matrices of the motifs for these proteins due to a limited number of sequences of the experimentally verified binding sites. For this reason, we

used an alternative approach in the assessment of the strength of binding sites (BSS) for the mentioned above splicing proteins, as proposed by Murray et al. [43]:

$$BSS = \frac{\sum_{i=0}^{i=L-k+1} \ln(4^k f_{n_i})}{L - k + 1},$$

where L is the length of the sequence of interest, k is the length of an oligomer (see below) found in the sequence of interest, f_n represents the frequency (within the set of sequences of the experimentally verified binding sites for a given splicing protein) of the oligomer found at the position i in the sequence of interest, and $\ln(4^k f_{n_i})$ is a log-odds representation of the degree to which the particular oligomer was enriched within the set of sequences of the experimentally verified binding sites for a given splicing protein. As proposed, we counted only the frequency of all the possible pentamers in the sequence of interest and used the frequency of pentamers from the set of sequences of the experimentally verified binding sites for a given splicing protein as the reference [43].

Bioinformatically predicted exonic splicing motifs. We selected three different approaches for de novo motifs discovery and identified 25 new motifs that were statistically associated with multi-spliced exons from the Ensembl database. First of all, we used the algorithm GADEM [44] from R/Bioconductor library rGADEM v.2.22.0 [45]. This approach was realized on a sub-set of multi-spliced exons with default settings of the software. The second approach was based on the heuristic algorithm bcrank from R/Bioconductor library BCRANK v.1.36.0 [46,47]. In this case, short sequences that were overrepresented in ranked exons (in descending order of their splicing degrees) were identified and the top motifs were selected for subsequent analysis. Finally, the algorithm motifRG from the R/Bioconductor library motifRG v.1.18.0 [41] was used with default settings. This algorithm searches for motifs that discriminate the given foreground and background sequences. We used a sub-set of multi-spliced exons as foreground sequences and other exons from our dataset as background sequences.

All newly identified motifs were converted into $\log_2$ position weight matrices with the correction against the background nucleotide frequency. Position weight matrices of Sironi's motifs 1-3 [48] were added to our collection of bioinformatically predicted exonic splicing motifs. Occurrence of these motifs in the sequence of interest was determined as described above. In addition, oligomers with the bioinformatically predicted exonic splicing activity were counted in the USE and DSE by the function vcountPDict from the R/Bioconductor library Biostrings v.2.42.0 [42] and their frequency was normalized relative to the length of the analysed sequences. The list of such oligomers included ESRE and ESRS hexamers [49], ESS decamers [50], PESE and PESS octamers [51,52], QUEPASA ESEseqs and QUEPASA ESSseqs hexamers [53] and RESCUE ESE hexamers [54].

Experimentally verified intronic splicing motifs. We could reconstruct the position weight matrices of the motifs for seven splicing proteins that bind intronic sequences for which we could not calculate the position weight matrices because of a limited number of sequences (Supplementary Data 11). We used the described above approaches for the determination of occurrence of all these motifs in the USIF and DSIF. It should be noted that some splicing proteins did not exhibit any intron/exon preference and are bound to both intronic and exonic motifs.

Bioinformatically predicted intronic splicing motifs. Oligomers with the bioinformatically predicted splicing activity were counted in the USIF and DSIF as described above. The list of such oligomers included Castle's oligomers [55], Culler's ISSs oligomers [56], Das' upstream and downstream intronic hexamers [57], Wang's ISEs hexamers [58], Wang's ISSs decamers [59], Yeo's downstream and upstream ISREs [60].

Polypyrimidine tract scoring. For each splicing event, the sequence of the polypyrimidine tract (-30 to -3 positions relative to the acceptor splice site) was extracted from the corresponding

DSIF. The strength of U2AF2 binding sites in this sequence was calculated according to Murray et al. [43]:

$$\mathrm{U2AF2_{strength}} = \frac{\sum_{i=0}^{i=L-k+1} \ln(4^k f_{n_i})}{L-k+1},$$

where L is the length of the extracted polypyrimidine tract sequence, k is the length of an oligomer found in the polypyrimidine tract sequence, f_n represents the frequency (within the U2AF2 selected SELEX sequences) of the oligomer found at the position i in the polypyrimidine tract sequence and $\ln\left(4^k f_{n_i}\right)$ is the log-odds representation of the degree to which a particular oligomer was enriched within the U2AF2 selected SELEX sequences [61]. This equation is identical to the equation for BSS, however, the values of independent variables in this equation allow us to work only with binding sites for the protein U2AF2. We counted the frequency of all the possible pentamers in the polypyrimidine tract and used the frequency of pentamers from the U2AF2 selected SELEX sequences as the reference [43].

Moreover, we collected G- and C-rich 4- to 7-nucleotide sequences overrepresented in intronic regions upstream of the weak polypyrimidine tracts [43]. We determined the frequency of these motifs in DSIF (-80 to -30 positions relative to the acceptor splice site) using the function vcountPDict from the R/Bioconductor library Biostrings v.2.42.0 [42] and normalized this parameter relative to the length of the analyzed intronic fragment.

Branchpoint sites scoring. First of all, we obtained genomic coordinates of 59,359 high-confidence human branchpoint sites from the work by Mercer et al. [62]. Next, we retrieved the 20-nucleotide sequences surrounding the branchpoints from the GRCh38/hg38 reference assembly of the Homo sapiens genome using the R/Bioconductor library BSgenome.Hsapiens.UCSC.hg38 [39] and used these sequences as a foreground set in the motif discovery analysis. Additionally, we developed a background set of sequences with 10-fold quantitative excess relative to the foreground set. This background set included randomly extracted 100-nucleotide sequences from the upstream regions of human introns.

Next, we used the discriminative motif discovery algorithm motifRG with default settings [41] and identified high confidence motif associated with human branchpoint sites. This motif was converted into $\log_2$ position weight matrix with the correction against the background nucleotide frequency and used for the scanning of the sequence of interest. For each splicing event, we extracted a 100-nucleotide sequence (-100 to -1 positions relative to the acceptor splice site) from the corresponding DSIF. For each extracted sequence, we calculated the maximal and mean motif affinity to the sequence of interest and a number of hits over the score threshold using the function motifScores from the R/Bioconductor library PWMEnrich v.4.10.0 [63]. As before, we used the 99th quantile of the motif weight distribution as a threshold in the identification of the true motif occurrence.

"Short" motifs. Frequency of x-mer (at $x \in [1,4]$) oligonucleotides in the sequence of interest was determined using the function oligonucleotideFrequency from the R/Bioconductor library Biostrings v.2.42.0 [42] and normalized relative to the length of the analyzed sequence.

Linear density of the minimal free energy of folding. The sequences of USE, USIF, DSIF, or DSE genomic/RNA elements were extracted from the GRCh38/hg38 reference assembly of the Homo sapiens genome using the R/Bioconductor library BSgenome.Hsapiens.UCSC.hg38 [39]. Free energy of folding, or minimal free energy (MFE), of these sequences was calculated using the RNAfold tool from ViennaRNA Package v.2.1.7 [64]. MFE was normalized relative to the length of the analyzed sequence and expressed as a linear density of the MFE [65,66].

Conservation scores. BW files with pre-computed conservation scores of the human GRCh38/hg38 reference genome were downloaded via the FTP server of the UCSC Genome Browser [67]. We used conservation scores that were calculated using the algorithms phyloP and phastCons after multiz-based multiple alignments of 99 vertebrate genomes to the

human genome [68,69]. Using the downloaded BW files, we calculated the minimum, maximum, standard deviation, and mean scores for each sequence of interest.

Functional features. Each splice site of EEJs was intersected with genomic coordinates of the splice sites of functionally grouped exons (see section 3.8). Next, exactly matched splice sites of EEJs were assigned to a functional class of respective exon.

Splicing distances. Splicing distances (length of introns) were directly retrieved from the matrix of the experimentally identified *EEJs*.

Size of exon clusters. This metric describes a distribution of constitutive and alternative splice sites along the body of the gene in the immediate vicinity of the splice site of interest. Exon clusters were calculated as described in section “Development a list of exon clusters”. The number of exons in a cluster was considered as the size of the cluster. Each splice site of EEJs was assigned the size of the exon cluster to which it and its partner splice site(-s) belonged.

Epigenetics features. In this study, we used data describing the location of five epigenetics marks in the genome of the siRR- or siMM-treated Kasumi-1 cells: CpGs islands, DNase I hypersensitivity sites, modified histone H3K9Ac, RNA polymerase II peaks and RUNX1/RUNX1T1 peaks. Distances of the splice sites of EEJs to the nearest epigenetic marks were measured using the function distanceToNearest from the R/Bioconductor library GenomicRanges v.1.32.3 [6].

Data mining with the random forest meta-classifier. Our primary data matrix included the class of exon-exon junction (differential or non-differential) as a dependent response variable and all the features described above as an independent predictor of variables. This matrix was filtered against the features that had only one unique value or features that had both characteristics: i) very few unique values relative to the number of samples and ii) the ratio of the frequency of the most common value to the frequency of the second most common value is large. Highly correlated features were removed with a cut-off 0.9 to reduce pair-wise correlations in the matrix. At this step, we used the functionality of the R library caret v.6.0-71 [70].

Feature importance. The importance of each feature was determined by calculating the total decrease in the node impurities from splitting on the feature averaged over all classification trees in random forest. The node impurity was measured with the Gini index. At this step, we used the R library randomForest v.4.6-12 in the classification mode [71,72]. We also used five independent runs of the random forest meta-classifier, and 1000 classification trees per random forest per run and ranked all features in descending order of importance.

Feature selection. We applied a recursive algorithm with five-fold cross-validation to select the minimal required set of important features. We used the function rfe from the R library caret v.6.0-71 at this step [70].

Final classification of EEJs. First of all, the data matrix was reduced to features selected in the previous step. Next, for each run of the random forest meta-classifier, the data matrix was randomly sampled on two sub-matrices: the training set (70% of input matrix) and the test set (30% of input matrix). We used the training set for machine learning, calculation of the proximity matrix and marginal effects of features. The test set was used to assess the classification accuracy. We used the R library randomForest v.4.6-12 in the classification mode at this step [71,72], 1,000 trees were grown at each algorithm run and the number of features sampled for splitting up at each node was equal to one third of all features in the input data matrix.

Reconstruction of the gene-regulatory networks. The sequences of promoter regions of the genes of interest were scanned against 2,287 position weight matrices of the human transcription factors from the MotifDb database [73]. To find out the true motifs in promoter sequences, lognormal threshold-free approach was used instead of the fixed-threshold algorithm. In this instance, transcription factors with significantly enriched motifs in the promoters of interest (enrichment score > 1 at $p < 0.05$ compared to a genomic background)

and with statistically significant differential expression ($p < 0.01$, FDR-adjusted $p < 0.1$) in the siRR- versus siMM-treated Kasumi-1 cells were selected for downstream analysis. Direct interactions of the fusion protein with the transcription factors genes and the target genes of interest were inferred from the ChIP-Seq data. Co-expression of RUNX1/RUNX1T1, genes coding transcription factors selected in the second step and the target genes of interest (genes encoding splicing factors and mRNA surveillance genes with differential expression) was inferred from the RNA-Seq and microarray data with CoExpress software [74]. From this analysis, only pairs of genes with more than 90% robust correlation in expression were selected. Genes with any discordance in expression and/or correlations between RNA-Seq and microarray data were excluded from the final list. In this instance, FDR of the correlations did not exceed 6.5% for the RNA-Seq data and 2.9% for the microarray data.

Gene enrichment analysis. We used up-to-date ODO and GAF files from the Gene Ontology Consortium [75,76] to develop a comprehensive list of the reference functional gene sets. From this list, we selected the gene sets containing ten or more members for downstream analysis. Next, two-sided Fisher's exact test was used to find out the under- and/or over-represented query gene set(-s) among the reference gene sets. Query results were parsed and under- or over-represented gene sets that passed members size ≥10 and FDR adjusted p-value threshold of 0.05 were collected. Finally, Cytoscape plug-in EnrichmentMap [77] was used to handle gene-set redundancy and hierarchical visualization of the enrichment results.

Gene set enrichment analysis. Gene set enrichment analysis was performed according to standard procedure using Molecular Signatures Database [78].

Assessment of the coding potential of RNA transcripts. Coding potential of RNA transcripts was assessed by on-line version of CPC2 software [79]. This software uses sequence features of transcripts and support vector machine for reliable prediction of coding ability of RNA molecules [80].

Identification of the significant open reading frames (ORFs) and premature termination codons (PTCs) in transcripts. Significant ORFs and PTCs were identified in the Cufflinks assembled transcripts as previously described [81]. In brief, all possible ATG-ORFs were identified in the transcript(s) of interest. Next, for each empirical transcript, 100 random sequences with the same length were generated using a multinomial model [82]. This new set of artificial transcripts was used to identify the ORFs. Finally, the 99th percentile of the distribution formed by the lengths of the artificial ORFs was used as a threshold for identification of the true ORF(s) in the empirical transcript. Transcripts with no significant ORFs were classified as non-coding. To identify PTCs, exonic structure and the coordinates of ORF(s) in the transcript of interest were matched. A transcript was annotated as PTC-containing, if the end of its ORF was localized ≥50 nucleotides upstream of the last exon-exon junction in the transcript.

Alignment classification of the in silico translated proteins. The *in silico* translated proteins were aligned against the human NCBI RefSeq proteins (release 92, last modified on 18 March 2019) and the newest non redundant releases of GenBank CDS translations, UniProtKB/SwissProt, Protein Data Bank, Protein Information Resource and Peptide/Protein Sequence Database proteins (updated on 11 April 2019) using NCBI blastp [83]. The *in silico* translated proteins with no alignment for any canonical proteins were classified as no hits. Next, according to identity value, all “with hits” proteins were divided into lowly identical (with identity below 90%) and highly identical (identity above 90%) to canonical proteins. Finally, all highly identical proteins were further classified into normal, N-extended, N-truncated, C-truncated, C-extended and complex based on the following criteria: i) normal, the *in silico* translated and reference proteins align perfectly; ii) N-extended, the *in silico* translated protein contains novel N-terminal amino acids followed by the full-length canonical protein sequence; iii) N-truncated, the *in silico* translated protein that lacks N-terminal part of the canonical

protein; iv) C-truncated, the *in silico* translated protein that lacks C-terminal part of the canonical protein; v) C-extended, the *in silico* translated protein that includes the full-length canonical protein sequence, followed by the novel C-terminal amino acids; vi) complex, the *in silico* translated protein other than canonical protein at both ends.

References

1 Morgan, M. *et al.* ShortRead: a bioconductor package for input, quality assessment and exploration of high-throughput sequence data. *Bioinformatics* **25**, 2607-2608, doi:10.1093/bioinformatics/btp450 (2009).

2 Speir, M. L. *et al.* The UCSC Genome Browser database: 2016 update. *Nucleic Acids Res* **44**, D717-725, doi:10.1093/nar/gkv1275 (2016).

3 Liao, Y., Smyth, G. K. & Shi, W. The Subread aligner: fast, accurate and scalable read mapping by seed-and-vote. *Nucleic acids research* **41**, e108-e108 (2013).

4 Morgan, M., Pages, H., Obenchain, V. & Hayden, N. Rsamtools: Binary alignment (BAM), FASTA, variant call (BCF), and tabix file import. *R package version* **1**, 677-689 (2016).

5 Lawrence, M. *et al.* Software for computing and annotating genomic ranges. *PLoS Comput Biol* **9**, e1003118, doi:10.1371/journal.pcbi.1003118 (2013).

6 Aboyoun, P., Pages, H. & Lawrence, M. Representation and manipulation of genomic intervals and variables defined along a genome. R package “GenomicRanges” version 1.32.3. (2018).

7 Carlson, M. *et al.* GenomicFeatures: Tools for making and manipulating transcript centric annotations. *R package version* **1** (2011).

8 Liao, Y., Smyth, G. K. & Shi, W. featureCounts: an efficient general purpose program for assigning sequence reads to genomic features. *Bioinformatics (Oxford, England)* **30**, 923-930, doi:10.1093/bioinformatics/btt656 (2014).

9 Derrien, T. *et al.* Fast computation and applications of genome mappability. *PloS one* **7**, e30377 (2012).

10 Boutz, P. L., Bhutkar, A. & Sharp, P. A. Detained introns are a novel, widespread class of post-transcriptionally spliced introns. *Genes & development* **29**, 63-80 (2015).

11 Braun, C. J. *et al.* Coordinated splicing of regulatory detained introns within oncogenic transcripts creates an exploitable vulnerability in malignant glioma. *Cancer cell* **32**, 411-426. e411 (2017).

12 Love, M. I., Huber, W. & Anders, S. Moderated estimation of fold change and dispersion for RNA-seq data with DESeq2. *Genome biology* **15**, 550 (2014).

13 Ritchie, M. E. *et al.* limma powers differential expression analyses for RNA-sequencing and microarray studies. *Nucleic acids research* **43**, e47-e47 (2015).

14 Robinson, M. D., McCarthy, D. J. & Smyth, G. K. edgeR: a Bioconductor package for differential expression analysis of digital gene expression data. *Bioinformatics* **26**, 139-140 (2010).

15 Anders, S., Reyes, A. & Huber, W. Detecting differential usage of exons from RNA-seq data. *Nature Precedings*, 1-1 (2012).

16 Love, M. I., Anders, S. & Huber, W. Differential gene expression analysis based on the negative binomial distribution. R package “DESeq2” version 1.20.0. (2018).

17 Anders, S. & Huber, W. Differential expression analysis for sequence count data. *Genome Biology* **11**, R106, doi:10.1186/gb-2010-11-10-r106 (2010).

18 Anders, S. *et al.* Count-based differential expression analysis of RNA sequencing data using R and Bioconductor. *Nature Protocols* **8**, 1765-1786, doi:10.1038/nprot.2013.099 (2013).

19 Robinson, M. D., McCarthy, D. J. & Smyth, G. K. edgeR: a Bioconductor package for differential expression analysis of digital gene expression data. *Bioinformatics (Oxford, England)* **26**, 139-140, doi:10.1093/bioinformatics/btp616 (2010).

20 Law, C. W., Chen, Y., Shi, W. & Smyth, G. K. voom: precision weights unlock linear model analysis tools for RNA-seq read counts. *Genome Biology* **15**, R29, doi:10.1186/gb-2014-15-2-r29 (2014).

21 Smyth, G. *et al.* Linear models for microarray data. R package “limma” version 3.36.2. (2018).

22 Benjamini, Y. & Hochberg, Y. Controlling the false discovery rate: A practical and powerful approach to multiple testing. *Journal of the Royal Statistical Society: Series B (Methodological)* **57**, 289-300, doi:10.1111/j.2517-6161.1995.tb02031.x (1995).

23 Hartley, S. W. & Mullikin, J. C. QoRTs: a comprehensive toolset for quality control and data processing of RNA-Seq experiments. *BMC bioinformatics* **16**, 224, doi:10.1186/s12859-015-0670-5 (2015).

24 Hartley, S. W. & Mullikin, J. C. Detection and visualization of differential splicing in RNA-Seq data with JunctionSeq. *Nucleic Acids Research* **44**, e127, doi:10.1093/nar/gkw501 (2016).

25 Roberts, A., Pimentel, H., Trapnell, C. & Pachter, L. Identification of novel transcripts in annotated genomes using RNA-Seq. *Bioinformatics* **27**, 2325-2329 (2011).

26 Trapnell, C. *et al.* Transcript assembly and abundance estimation from RNA-Seq reveals thousands of new transcripts and switching among isoforms. *Nature biotechnology* **28**, 511-515, doi:10.1038/nbt.1621 (2010).

27 Trapnell, C. *et al.* Differential gene and transcript expression analysis of RNA-seq experiments with TopHat and Cufflinks. *Nature Protocols* **7**, 562-578, doi:10.1038/nprot.2012.016 (2012).

28 Trapnell, C. *et al.* Differential analysis of gene regulation at transcript resolution with RNA-seq. *Nature Biotechnology* **31**, 46-53, doi:10.1038/nbt.2450 (2013).

29 Lawrence, M. *et al.* Software for computing and annotating genomic ranges. *PLoS computational biology* **9**, e1003118, doi:10.1371/journal.pcbi.1003118 (2013).

30 Chen, Y. *et al.* Empirical analysis of digital gene expression data in R. R package “edgeR” version 3.22.3. (2018).

31 Eslami, A., Qannari, E. M., Bougeard, S. & Sanchez, G. Title multigroup data analysis. R package “multigroup” version 0.4.0. (2015).

32 Karatzoglou, A., Smola, A., Hornik, K. & Zeileis, A. kernlab - An S4 Package for Kernel Methods in R. *Journal of Statistical Software* **11**, 1-20 (2004).

33 Krijthe, J. & van der Maaten, L. J. P. T-distributed stochastic neighbor embedding using a Barnes-Hut implementation. R package “Rtsne” version 0.13. (2017).

34 Maaten, L. v. d. Accelerating t-SNE using tree-based algorithms. **15**, 3221–3245 (2014).

35 Hyvärinen, A. & Oja, E. Independent component analysis: algorithms and applications. *Neural Networks: The Official Journal of the International Neural Network Society* **13**, 411-430 (2000).

36 Nazarov, P. V. *et al.* Deconvolution of transcriptomes and miRNomes by independent component analysis provides insights into biological processes and clinical outcomes of melanoma patients. *BMC medical genomics* **12**, 132 (2019).

37 Ptasinska, A. *et al.* Depletion of RUNX1/ETO in t(8;21) AML cells leads to genome-wide changes in chromatin structure and transcription factor binding. *Leukemia* **26**, 1829-1841, doi:10.1038/leu.2012.49 (2012).

38 Cairns, J. *et al.* BayesPeak-an R package for analysing ChIP-seq data. *Bioinformatics* **27**, 713-714, doi:10.1093/bioinformatics/btq685 (2011).

39 Team, T. B. D. BSgenome.Hsapiens.UCSC.hg38: Full genome sequences for Homo sapiens (UCSC version hg38). R package "BSgenome.Hsapiens.UCSC.hg38" version 1.4.1. (2015).

40 Yeo, G., Holste, D., Kreiman, G. & Burge, C. B. Variation in alternative splicing across human tissues. *Genome Biology* **5**, R74, doi:10.1186/gb-2004-5-10-r74 (2004).

41 Yao, Z. *et al.* Discriminative motif analysis of high-throughput dataset. *Bioinformatics (Oxford, England)* **30**, 775-783, doi:10.1093/bioinformatics/btt615 (2014).
42 Pagès, H., Aboyoun, P., Gentleman, R. & DebRoy, S. Biostrings: String objects representing biological sequences, and matching algorithms. R package version 2.42.0. (2014).
43 Murray, J. I., Voelker, R. B., Henscheid, K. L., Warf, M. B. & Berglund, J. A. Identification of motifs that function in the splicing of non-canonical introns. *Genome Biology* **9**, R97, doi:10.1186/gb-2008-9-6-r97 (2008).
44 Li, L. GADEM: a genetic algorithm guided formation of spaced dyads coupled with an EM algorithm for motif discovery. *Journal of Computational Biology: A Journal of Computational Molecular Cell Biology* **16**, 317-329, doi:10.1089/cmb.2008.16TT (2009).
45 Droit, A., Gottardo, R., Robertson, G. & Li, L. rGADEM: de novo motif discovery. R package "rGADEM" version 2.22.0. (2014).
46 Ameur, A. BCRANK: Predicting binding site consensus from ranked DNA sequences. R package version 1.40.0. (2010).
47 Ameur, A., Rada-Iglesias, A., Komorowski, J. & Wadelius, C. Identification of candidate regulatory SNPs by combination of transcription-factor-binding site prediction, SNP genotyping and haploChIP. *Nucleic Acids Research* **37**, e85, doi:10.1093/nar/gkp381 (2009).
48 Sironi, M. *et al.* Silencer elements as possible inhibitors of pseudoexon splicing. *Nucleic Acids Research* **32**, 1783-1791, doi:10.1093/nar/gkh341 (2004).
49 Goren, A. *et al.* Comparative analysis identifies exonic splicing regulatory sequences-The complex definition of enhancers and silencers. *Molecular Cell* **22**, 769-781, doi:10.1016/j.molcel.2006.05.008 (2006).
50 Wang, Z. *et al.* Systematic identification and analysis of exonic splicing silencers. *Cell* **119**, 831-845, doi:10.1016/j.cell.2004.11.010 (2004).
51 Zhang, X. H.-F. & Chasin, L. A. Computational definition of sequence motifs governing constitutive exon splicing. *Genes & Development* **18**, 1241-1250, doi:10.1101/gad.1195304 (2004).
52 Zhang, X. H.-F., Kangsamaksin, T., Chao, M. S. P., Banerjee, J. K. & Chasin, L. A. Exon inclusion is dependent on predictable exonic splicing enhancers. *Molecular and Cellular Biology* **25**, 7323-7332, doi:10.1128/MCB.25.16.7323-7332.2005 (2005).
53 Ke, S. *et al.* Quantitative evaluation of all hexamers as exonic splicing elements. *Genome Research* **21**, 1360-1374, doi:10.1101/gr.119628.110 (2011).
54 Fairbrother, W. G., Yeh, R.-F., Sharp, P. A. & Burge, C. B. Predictive identification of exonic splicing enhancers in human genes. *Science (New York, N.Y.)* **297**, 1007-1013, doi:10.1126/science.1073774 (2002).
55 Castle, J. C. *et al.* Expression of 24,426 human alternative splicing events and predicted *cis* regulation in 48 tissues and cell lines. *Nature Genetics* **40**, 1416-1425, doi:10.1038/ng.264 (2008).
56 Culler, S. J., Hoff, K. G., Voelker, R. B., Berglund, J. A. & Smolke, C. D. Functional selection and systematic analysis of intronic splicing elements identify active sequence motifs and associated splicing factors. *Nucleic Acids Research* **38**, 5152-5165, doi:10.1093/nar/gkq248 (2010).
57 Das, R. *et al.* SR proteins function in coupling RNAP II transcription to pre-mRNA splicing. *Molecular Cell* **26**, 867-881, doi:10.1016/j.molcel.2007.05.036 (2007).
58 Wang, Y., Ma, M., Xiao, X. & Wang, Z. Intronic splicing enhancers, cognate splicing factors and context-dependent regulation rules. *Nature Structural & Molecular Biology* **19**, 1044-1052, doi:10.1038/nsmb.2377 (2012).
59 Wang, Y. *et al.* A complex network of factors with overlapping affinities represses splicing through intronic elements. *Nature Structural & Molecular Biology* **20**, 36-45, doi:10.1038/nsmb.2459 (2013).

60 Yeo, G. W., Van Nostrand, E. L., Nostrand, E. L. V. & Liang, T. Y. Discovery and analysis of evolutionarily conserved intronic splicing regulatory elements. *PLoS genetics* **3**, e85, doi:10.1371/journal.pgen.0030085 (2007).
61 Banerjee, H. *et al.* The conserved RNA recognition motif 3 of U2 snRNA auxiliary factor (U2AF 65) is essential in vivo but dispensable for activity in vitro. *RNA (New York, N.Y.)* **10**, 240-253 (2004).
62 Mercer, T. R. *et al.* Genome-wide discovery of human splicing branchpoints. *Genome Research* **25**, 290-303, doi:10.1101/gr.182899.114 (2015).
63 Stojnic, R. & Diez, D. PWMEnrich: PWM enrichment analysis. R package version 4.10.0. (2015).
64 Lorenz, R. *et al.* ViennaRNA Package 2.0. *Algorithms for Molecular Biology* **6**, 26, doi:10.1186/1748-7188-6-26 (2011).
65 Pervouchine, D. D., Graber, J. H. & Kasif, S. On the normalization of RNA equilibrium free energy to the length of the sequence. *Nucleic Acids Research* **31**, e49 (2003).
66 Posrednik, D. V. *et al.* [Structural and thermodynamic features of intergenic and intronic human primary microRNAs]. *Molekuliarnaia Biologiia* **45**, 554-564 (2011).
67 Harrow, J. L. *et al.* The Vertebrate Genome Annotation browser 10 years on. *Nucleic Acids Research* **42**, D771-779, doi:10.1093/nar/gkt1241 (2014).
68 Pollard, K. S., Hubisz, M. J., Rosenbloom, K. R. & Siepel, A. Detection of nonneutral substitution rates on mammalian phylogenies. *Genome Research* **20**, 110-121, doi:10.1101/gr.097857.109 (2010).
69 Siepel, A. *et al.* Evolutionarily conserved elements in vertebrate, insect, worm, and yeast genomes. *Genome Research* **15**, 1034-1050, doi:10.1101/gr.3715005 (2005).
70 Kuhn, M. caret: Classification and regression training. R package "caret" version 6.0-71. (2016).
71 Breiman and Cutler’s random forests for classification and regression. R package “randomForest” version 4.6-12. (2016).
72 Liaw, A. & Wiener, M. Classification and regression by randomForest. R News 2:18-22 (2002).
73 Shannon, P. & Richards, M. MotifDb: An annotated collection of protein-DNA binding sequence motifs. (2018).
74 Nazarov, P. V. *et al.* Interplay of microRNAs, transcription factors and target genes: linking dynamic expression changes to function. *Nucleic Acids Research* **41**, 2817-2831, doi:10.1093/nar/gks1471 (2013).
75 Ashburner, M. *et al.* Gene ontology: tool for the unification of biology. The Gene Ontology Consortium. *Nature Genetics* **25**, 25-29, doi:10.1038/75556 (2000).
76 The Gene Ontology Consortium. Expansion of the Gene Ontology knowledgebase and resources. *Nucleic Acids Research* **45**, D331-D338, doi:10.1093/nar/gkw1108 (2017).
77 Merico, D., Isserlin, R., Stueker, O., Emili, A. & Bader, G. D. Enrichment map: a network-based method for gene-set enrichment visualization and interpretation. *PloS One* **5**, e13984, doi:10.1371/journal.pone.0013984 (2010).
78 Subramanian, A. *et al.* Gene set enrichment analysis: a knowledge-based approach for interpreting genome-wide expression profiles. *Proceedings of the National Academy of Sciences of the United States of America* **102**, 15545-15550, doi:10.1073/pnas.0506580102 (2005).
79 Kang, Y.-J. *et al.* CPC2: a fast and accurate coding potential calculator based on sequence intrinsic features. *Nucleic Acids Research* **45**, W12-W16, doi:10.1093/nar/gkx428 (2017).
80 Kong, L. *et al.* CPC: assess the protein-coding potential of transcripts using sequence features and support vector machine. *Nucleic Acids Research* **35**, W345-349, doi:10.1093/nar/gkm391 (2007).

81 Grinev, V. V. *et al.* Decoding of exon splicing patterns in the human RUNX1-RUNX1T1 fusion gene. *The International Journal of Biochemistry & Cell Biology* **68**, 48-58, doi:10.1016/j.biocel.2015.08.017 (2015).

82 Ababneh, F., Jermiin, L. S. & Robinson, J. Generation of the Exact Distribution and Simulation of Matched Nucleotide Sequences on a Phylogenetic Tree. *Journal of Mathematical Modelling and Algorithms* **5**, 291-308, doi:10.1007/s10852-005-9017-y (2006).

83 Gish, W. & States, D. J. Identification of protein coding regions by database similarity search. *Nature Genetics* **3**, 266-272, doi:10.1038/ng0393-266 (1993).
