# Supplementary material for: RUNX1/RUNX1T1 mediates alternative splicing and reorganises the transcriptional landscape in leukemia
Source: Nat Commun. 2021 Jan 22;12:520. doi: 10.1038/s41467-020-20848-z (PMC7822815; doi:10.1038/s41467-020-20848-z)
Supplement: Supplementary file 3 — Description of Additional Supplementary Files [file 41467_2020_20848_MOESM3_ESM.pdf]

Description of Additional Supplementary Files

File Name: Supplementary Data 1
Description: Brief description of data sets used in this study

File Name: Supplementary Data 2
Description: DAVID 6.8 gene sets that are significantly (p-value < 0.00004, q-value < 0.05) overrepresented among the direct targets of RUNX1/RUNX1T1

File Name: Supplementary Data 3
Description: Complete list of direct target genes of RUNX1/RUNX1T1 fusion protein in siMM-treated Kasumi-1 cells

File Name: Supplementary Data 4
Description: **.** List of genes coding splicing factors and mRNA surveillance genes that differentially expressed (p < 0.01, q < 0.1) in siRR versus siMM treated Kasumi-1 cells

File Name: Supplementary Data 5
Description: List of retained introns detected in the transcriptome of Kasumi-1 cells

File Name: Supplementary Data 6
Description: List of the differentially used exons (more than 2-fold change, p-value < 0.0005, q-value < 0.1) detected in the transcriptome of Kasumi-1 cells following *RUNX1/RUNX1T1* knockdown

File Name: Supplementary Data 7
Description: List of the differential exon-exon junctions (more than 2-fold change, p-value < 0.0003, q-value < 0.1) detected in the transcriptome of Kasumi-1 cells following *RUNX1/RUNX1T1* knockdown

File Name: Supplementary Data 8
Description: qPCR-based validation of differential splicing at the level of exon-exon junctions in the transcriptome of Kasumi-1 cells following *RUNX1/RUNX1T1* knockdown

File Name: Supplementary Data 9
Description: Summary of the independent component analysis results for primary t(8;21)-positive AML compared to normal CD34-positive cells

File Name: Supplementary Data 10
Description: Summary of the independent component analysis results for primary t(8;21)-positive AML compared to other AML subtypes

File Name: Supplementary Data 11
Description: List of genes coding splicing factors and mRNA surveillance genes that differentially expressed (p < 0.01, q < 0.1) in siRR versus siMM treated Kasumi-1 cells

File Name: Supplementary Data 12
Description: List of features associated with EEJs. Features significantly associated with differential splicing are highlighted in sky blue

File Name: Supplementary Data 13
Description: Extended motif analysis of splicing factors associated with differential splicing in the transcriptome of Kasumi-1 cells following RUNX1/RUNX1T1 knockdown

File Name: Supplementary Data 14
Description: List of primers for the qPCR-based analysis of gene expression

File Name: Supplementary Data 15
Description: List of references on exonic and intronic motifs for the splicing factors
