# Supplementary material for: RUNX1/RUNX1T1 mediates alternative splicing and reorganises the transcriptional landscape in leukemia
Source: Nat Commun. 2021 Jan 22;12:520. doi: 10.1038/s41467-020-20848-z (PMC7822815; doi:10.1038/s41467-020-20848-z)
Supplement: Supplementary file 19 — Reporting Summary [file 41467_2020_20848_MOESM19_ESM.pdf]

nature research

Corresponding author(s): Vasily Grinev, Olaf Heidenreich

Last updated by author(s): 15-11-2020

Reporting Summary

Nature Research wishes to improve the reproducibility of the work that we publish. This form provides structure for consistency and transparency in reporting. For further information on Nature Research policies, see our Editorial Policies and the Editorial Policy Checklist.

Statistics

For all statistical analyses, confirm that the following items are present in the figure legend, table legend, main text, or Methods section.

n/a	Confirmed	
☐	☒	The exact sample size (*n*) for each experimental group/condition, given as a discrete number and unit of measurement
☐	☒	A statement on whether measurements were taken from distinct samples or whether the same sample was measured repeatedly
☐	☒	The statistical test(s) used AND whether they are one- or two-sided<br>*Only common tests should be described solely by name; describe more complex techniques in the Methods section.*
☐	☒	A description of all covariates tested
☐	☒	A description of any assumptions or corrections, such as tests of normality and adjustment for multiple comparisons
☐	☒	A full description of the statistical parameters including central tendency (e.g. means) or other basic estimates (e.g. regression coefficient) AND variation (e.g. standard deviation) or associated estimates of uncertainty (e.g. confidence intervals)
☐	☒	For null hypothesis testing, the test statistic (e.g. *F*, *t*, *r*) with confidence intervals, effect sizes, degrees of freedom and *P* value noted<br>*Give P values as exact values whenever suitable.*
☐	☒	For Bayesian analysis, information on the choice of priors and Markov chain Monte Carlo settings
☐	☒	For hierarchical and complex designs, identification of the appropriate level for tests and full reporting of outcomes
☐	☒	Estimates of effect sizes (e.g. Cohen's *d*, Pearson's *r*), indicating how they were calculated

Our web collection on statistics for biologists contains articles on many of the points above.

Software and code

Policy information about availability of computer code

Data collection	na
Data analysis	All in-laboratory developed source codes are available at GitHub project https://github.com/VGrinev/TranscriptomicFeatures

For manuscripts utilizing custom algorithms or software that are central to the research but not yet described in published literature, software must be made available to editors and reviewers. We strongly encourage code deposition in a community repository (e.g. GitHub). See the Nature Research guidelines for submitting code & software for further information.

Data

Policy information about availability of data

All manuscripts must include a data availability statement. This statement should provide the following information, where applicable:

- Accession codes, unique identifiers, or web links for publicly available datasets
- A list of figures that have associated raw data
- A description of any restrictions on data availability

The accession numbers for all the microarray and next generation sequencing data reported in this paper is listed in Supplementary Data 1.

Field-specific reporting

Life sciences study design

All studies must disclose on these points even when the disclosure is negative.

Sample size	Three biological replicates were chosen as the minimum sample size, and the necessary statistics were carried out on them.
Data exclusions	No data exclusion was performed in the study.
Replication	We performed each experiment at least three times to ensure data reproducibility. The exact number of times each experiment was performed is stated in the text or corresponding figure .
Randomization	No randomization was performed in the study.
Blinding	No blinding was performed in the study.

Reporting for specific materials, systems and methods

We require information from authors about some types of materials, experimental systems and methods used in many studies. Here, indicate whether each material, system or method listed is relevant to your study. If you are not sure if a list item applies to your research, read the appropriate section before selecting a response.

Materials & experimental systems

n/a	Involved in the study	
☐	☒	Antibodies
☐	☒	Eukaryotic cell lines
☒	☐	Palaeontology and archaeology
☒	☐	Animals and other organisms
☒	☐	Human research participants
☒	☐	Clinical data
☒	☐	Dual use research of concern

Methods

n/a	Involved in the study	
☒	☐	ChIP-seq
☒	☐	Flow cytometry
☒	☐	MRI-based neuroimaging

Antibodies

Antibodies used	The following antibodies were used in this study: rabbit polyclonal anti-RUNX1 (cat. #4334S, Cell Signaling Technology), rabbit monoclonal anti-RPS6KA1 (cat. #8408S, Cell Signaling Technology), mouse monoclonal anti-LIMS1 (cat. #11890S, Cell Signaling Technology), mouse monoclonal anti-GAPDH (cat. #AM4300, InvitrogenTM), rabbit polyclonal anti-PARL #1 (Thomas Langer' laboratory), rabbit polyclonal anti-PARL #2 (cat. #ab45231, Abcam), goat polyclonal anti-mouse (cat. #P0447, Agilent), goat polyclonal anti-rabbit (cat. #sc-2004, Santa Cruz Biotechnology) antibodies.
Validation	Validation of all commercial antibodies can be found on the manufacturer's website using the provided catalog number. The rabbit polyclonal anti-PARL antibodies #1 were developed and validated in Thomas Langer' laboratory.

Eukaryotic cell lines

Policy information about cell lines

Cell line source(s)	Kasumi-1 (DSMZ no. ACC 220), SKNO-1 (DSMZ no. ACC 690).
Authentication	Cell authentication was performed on regular basis.
Mycoplasma contamination	All the cells used in the study were mycoplasma negative.
Commonly misidentified lines (See ICLAC register)	No commonly misidentified cell lines were used in the study.
